# Oxidized alginate hydrogels as niche environments for corneal epithelial cells

**DOI:** 10.1002/jbm.a.35011

**Published:** 2013-10-28

**Authors:** Bernice Wright, Paul A De Bank, Kim A Luetchford, Fernando R Acosta, Che J Connon

**Affiliations:** 1 School of Chemistry, Food and Pharmacy, Department of Pharmaceutics, University of ReadingReading, Berkshire, RG6 6UB, United Kingdom; 2 Department of Pharmacy and Pharmacology, University of BathBath, BA2 7AY, United Kingdom; 3 Department of Chemical Engineering, University of BathBath, BA2 7AY, United Kingdom

**Keywords:** oxidised alginate, limbal epithelial cells, limbal stem cell deficiency, alginate gel stiffness, alginate gel porosity

## Abstract

Chemical and biochemical modification of hydrogels is one strategy to create physiological constructs that maintain cell function. The aim of this study was to apply oxidised alginate hydrogels as a basis for development of a biomimetic niche for limbal epithelial stem cells that may be applied to treating corneal dysfunction. The stem phenotype of bovine limbal epithelial cells (LEC) and the viability of corneal epithelial cells (CEC) were examined in oxidised alginate gels containing collagen IV over a 3-day culture period. Oxidation increased cell viability (*P* ≤ 0.05) and this improved further with addition of collagen IV (*P* ≤ 0.01). Oxidised gels presented larger internal pores (diameter: 0.2–0.8 µm) than unmodified gels (pore diameter: 0.05–0.1 µm) and were significantly less stiff (*P* ≤ 0.001), indicating that an increase in pore size and a decrease in stiffness contributed to improved cell viability. The diffusion of collagen IV from oxidised alginate gels was similar to that of unmodified gels suggesting that oxidation may not affect the retention of extracellular matrix proteins in alginate gels. These data demonstrate that oxidised alginate gels containing corneal extracellular matrix proteins can influence corneal epithelial cell function in a manner that may impact beneficially on corneal wound healing therapy. © 2013 The Authors. Journal of Biomedical Materials Research Part A Published byWiley Periodicals, Inc. Part A: 102A: 3393–3400, 2014.

## INTRODUCTION

Limbal epithelial stem cells (LESC) are one of a small group of progenitor cells that are currently applied as a successful clinical therapy. LESC are resident adult stem cells in the outer edge of the cornea that terminally differentiate into limbal epithelial cells (LEC), which migrate across the ocular surface finally forming the uppermost layer of the cornea before desquamation. These cells, particularly when supported upon amniotic membrane (AM) scaffolds, can reverse corneal blindness caused by limbal stem cell deficiency (LSCD).^1^–^3^ A key issue associated with the therapeutic use of LESC, however, is the variation in clinical outcomes following their transplantation, due to differences in the severity of injuries from patient to patient. Another problem is that the AM scaffold is heterogeneous in structure and this affects LESC culture.^2^,^4^,^5^ AM preparation is also time-consuming and involves serological screening to monitor the risk of viral transmission.

These problems may be eliminated through the refinement of existing LSCD therapy by using structurally modified biomaterials to deliver LESC.^6^ Advantages of biomaterials include structural uniformity and malleability, predictable degradation, and the ability to direct stem cell fate. These matrices can, therefore, allow a number of different strategies to address variations in the severity of corneal injuries, including delivery of greater numbers of LESC to the cornea, more efficient maintenance of the LESC phenotype, and delivery of LESC-derived biomolecules.^7^ Native biomaterial constructs are not sufficient to maintain the physiological functions and phenotype of cells as they do not present a normal *in vivo* environment. These matrices often require biophysical (e.g., increasing internal pore size, decreasing compressive modulus/stiffness) and biochemical (e.g., cell adhesion peptide motifs, extracellular matrix (ECM) proteins) modifications to present the cells with conditions which support their ‘normal' function. Therefore, the addition of niche-like properties (e.g., inclusion of ECM proteins or cell adhesion peptides and modification of porosity to mimic physiological micro fluidic features) into existing biomaterial structures is increasingly employed. The aim of the present study was to investigate oxidised alginate hydrogels containing the corneal basement membrane ECM protein collagen IV, as matrices for the encapsulation of corneal epithelial cells in order to begin construction of artificial niche-like environments applicable to therapeutic delivery of these cells.

Alginate is well-established as a biocompatible material for cell encapsulation^8^–^10^ and tissue transplantation,^11^,^12^ but modifications to native alginate hydrogels to create gels that mimic the physiological environment of encapsulated cells are necessary to allow optimum cell viability and function.^13^,^14^ We have previously investigated alginate hydrogels as substrates for storage/transport of LESC and LEC,^8^,^9^ and found that increasing the porosity and decreasing the stiffness of alginate gels increased the viability of encapsulated cells.^8^ Other studies have shown that the viscoelastic^15^ and physicochemical^16^ properties of alginate gels and their chemical modification (e.g., sulfation,^17^ oxidation^18^–^24^) can direct cell function.

Oxidised alginate gels in particular were previously demonstrated to be cyto-compatible for a number of cell types including corneal endothelial cells,^18^ hepatocytes,^19^ fibroblasts,^20^ mesenchymal stem cells,^21^ adipose stem cells,^22^ and human osteo-progenitors.^23^ Studies have been performed to investigate the use of oxidised alginate as a wound healing scaffold.^24^,^25^ Oxidation of alginate affects the swelling behavior, degradation profile, and storage modulus of the resultant gel, allowing these features to be controlled depending on the degree of oxidation. It is these properties that underpin the beneficial wound healing effects of alginate scaffolds.^22^,^23^ Predictable degradation of alginate gels is an important structural feature that can allow the controlled release of therapeutic biomolecules or cells into damaged tissues. Tissue engineering strategies are currently under investigation using degradable oxidised alginate hydrogels—these matrices were shown to greatly improve cartilage-like tissue formation *in vivo*^23^ as well as the growth of adipose tissue from adipose stem cells.^22^ The possibility of introducing proteins into oxidised alginate gel systems to create an extracellular matrix-like milieu has also been explored. Recent reports have described oxidized RGD–alginate^23^,^26^ and oxidised alginate crosslinked with gelatin as efficient wound healing scaffolds.^24^

In the present study we demonstrate that the viability of both bovine primary and immortalised human corneal epithelial cells [HCE: referred to as corneal epithelial cells (CEC)] immobilized in alginate gels was enhanced by prior oxidation of these matrices. We also show that incorporating collagen IV, a key ECM protein in the corneal basement membrane, into oxidised gels caused a further enhancement in cell viability. We correlated increased viability to an increase in porosity and a decrease in gel stiffness and observed that these gels were able to support a population of undifferentiated LEC. We conclude that oxidised alginate gels containing limbal ECM proteins may be developed into important tools for regeneration of the injured or dysfunctional cornea. For example, a niche-like environment formed from modified alginate gels containing LESC may allow these cells to be engrafted without expansion, then to either migrate from the gel or secrete factors that allow repair of the damaged ocular surface.

## MATERIALS AND METHODS

### Materials

Dulbecco's minimal essential medium (DMEM) and Ham's F12 medium (DMEM/F12,1:1), antibiotics (penicillin/streptomycin), fetal bovine serum (FBS), amphotericin B, dimethylsulfoxide (DMSO), HCl, Whatman chromatography paper, O.C.T (TissueTek) and Trypan blue were purchased from Fisher Scientific (Leicestershire, UK). Human epidermal growth factor (hEGF), insulin, sodium alginate (viscosity: 15–20 cP, 1% in H_2_O (lit.)), calcium chloride, 3-(4,5-dimethylthiazol-2-yl)-2,5-diphenyltetrazolium bromide (MTT reagent), sodium citrate, sodium chloride, sodium periodate, ethylene glycol, starch solution, potassium iodide, sodium phosphate buffer and ethyl alcohol were from Sigma–Aldrich (Poole, UK). The HCE cell-line (CEC) was from RIKEN BioResource Center (Tsukuba, Ibaraki, Japan). Osmium tetroxide was from Agar Scientific (Essex, UK). The enhanced chemiluminescence (ECL) detection system and modified Lowry protein assay kit were obtained from Pierce (Thermo Fisher Scientific; Rockford, IL). The 14-3-3ζ antibody (rabbit polyclonal, immunogen: human 14-3-3ζ peptide) was from Santa Cruz Biotechnology (Autogen Bioclear UK, Calne, Wilts, UK), the cytokeratin 3 (CK3: clone AE5—mouse monoclonal, immunogen: rabbit corneal epithelial keratin) antibody was purchased from Millipore (Dundee, Scotland) and the cytokeratin 14 [CK14: guinea pig polyclonal, immunogen: Recombinant human keratin K14 (complete polypeptide)] antibody was from Progen Scientific (Merton, UK). Horseradish peroxidase (HRP)-conjugated secondary antibodies (anti-mouse (1:8,000 dilution) and anti-guinea pig (1:10,000 dilution)) were from R and D systems (Abingdon, UK) and bovine serum albumin (BSA) was obtained from First Link (Birmingham, UK).

### Methods

#### Oxidation of alginate

A 1% (w/v) solution of sodium alginate (8.0 g, 40.4 mmol uronate) in distilled water was mixed with an aqueous solution of sodium periodate (0.25*M*, 2.0 mmol) and incubated at room temperature for 24 h, after which the reaction was quenched by the addition of an equimolar amount of ethylene glycol. Sodium chloride (20 g) was then added to the solution, followed by precipitation with 2 excess volumes of ethyl alcohol (1.6 L). The precipitates, collected by centrifugation, were redissolved in distilled water (0.8 L) and precipitated again with ethyl alcohol (1.6 L). The resulting oxidized alginate was freeze-dried (Manufacturer: Thermo Scientific, Model: MicroModulyo 230) under reduced pressure to yield a white solid (7.2 g, 90% yield).

#### Determination of degree of oxidation

The degree of alginate oxidation was determined by measuring the percentage of sodium periodate consumed before the reaction was quenched with ethylene glycol. The consumption of sodium periodate was monitored spectrophotometrically using starch as an indicator. Briefly, equal volumes of freshly prepared potassium iodide solution (20% (w/v), pH 7.0 sodium phosphate buffer) and starch solution (10% (w/v), pH 7.0 sodium phosphate buffer) were mixed and immediately reacted with a solution of the oxidized alginate at room temperature. The concentration of the unreacted periodate was measured spectrophotometrically at 486 nm and this value was used to determine the amount of periodate consumed.

#### Determination of the molecular weight of oxidised alginate by size exclusion chromatography

Average molecular weights were determined by gel permeation chromatography using a Shimadzu HPLC system with RID-10A refractive index detector and SEC TSK G3000PW and G4000PWxl columns (Phenomenex, Mcclesfield, Cheshire UK). Samples were run at 27°C in ultrapure water.

#### Isolation of limbal epithelial cells from the cornea

The established bovine cornea model^4^,^27^,^28^ was used for the isolation of limbal epithelial cells (LEC). Normal bovine eyes were obtained from a local abattoir (Chitty Wholesale abattoir, Guildford, UK) within 2 h of death, transported to the laboratory at 4°C and used immediately. Corneoscleral rims were dissected using standard eye bank techniques, as previously described.^4^,^27^,^28^ Primary LEC were not cultured prior to encapsulation in alginate gels, they were encapsulated immediately following isolation; these cells were incorporated in this study to examine the effect of alginate oxidation on their progenitor phenotype.

#### Culture of corneal epithelial cells

A human corneal epithelial cell-line^29^ (CEC) was cultured in DMEM/F12, 1:1 supplemented with 5% (v/v) FBS, 0.5% (v/v) DMSO, 10 ng mL^−1^ hEGF, 5 mg mL^−1^ insulin, 100 IU mL^−1^ penicillin and 100 mg mL^−1^ streptomycin, at 37°C under 5% CO_2_ and 95% humidity. Cells were replenished with fresh medium every 3 days and grown to 70–80% confluency before encapsulation in calcium alginate gel discs. CEC were used in the present study as proof of concept to determine the biocompatibility of oxidised alginate gels for these corneal cells.

#### Encapsulation of CEC and LEC in calcium alginate gel discs

CEC or primary LEC (3 × 10^5^ cells) were suspended in 1.2% (w/v) sodium alginate solution with or without collagen IV (1 mg mL^−1^) before gelling into discs^6^ using 102 m*M* CaCl_2_. To form gel discs, a paper ring with a 2 cm diameter opening was placed over a 3 cm diameter paper disc, and saturated with 102 m*M* CaCl_2_ before unmodified or oxidised alginate solution (420 µL) was pipetted into the ring. A second 3-cm diameter paper disc saturated with 102 m*M* CaCl_2_ was placed over the alginate/paper assembly. Alginate solutions were exposed to 102 m*M* CaCl_2_ for 10 min to allow complete gelation. Calcium alginate discs with or without cells were suspended in DMEM/F12 medium under cell culture (37°C, 5% CO_2_, 95% humidity) conditions for 3 days. Cells were extracted from gel discs using alginate dissolving buffer (0.15*M* NaCl, 0.055*M* sodium citrate).

#### Cell viability analysis

The Trypan blue exclusion assay was performed by mixing a 10 µL cell suspension with 10 µL Trypan blue dye solution, before counting live (unstained) and dead (stained-blue) cells using a haemocytometer.

#### Immunoblotting

Sodium dodecyl sulfate–polyacrylamide gel electrophoresis (SDS-PAGE) was carried out in discontinuous vertical slab gels which contained a final concentration of 15% v/v acrylamide in the resolving gel and 4% (v/v) acrylamide in the stacking gel. The stacking gel contained stacking gel buffer (0.5*M* Tris-base; pH 6.8), 30% (v/v) acrylamide, 10% (w/v) SDS, 0.05% (v/v) TEMED and 1.5% (w/v) AMPS. The resolving gel comprised resolving gel buffer (3*M* Tris-base and 5*N* HCl; pH 8.8), 30% (v/v) acrylamide, 10% (w/v) SDS, 0.08% (v/v) TEMED and 1.5% (w/v) AMPS. The amount of total protein in LESC lysates and CM samples was quantified using the modified Lowry protein assay, and samples were prepared by addition of an equal volume of Laemmli reducing sample-treatment buffer (RSTB: 4% (w/v) SDS, 10% (v/v) 2-mercaptoethanol, 20% (v/v) glycerol, 0.5 M Tris-base and 0.001% (w/v) bromophenol blue) to solutions of lysed cells and CM (1:1). RSTB-treated samples (10 µg total protein per sample) were loaded onto the stacking gel and separated in resolving gels using 100 V per gel. Gels were then immunoblotted.

Proteins were transferred from SDS-PAGE gels to PVDF membranes under semidry conditions using 200 V per gel for 2 h. Nonspecific binding to membranes was blocked by incubation with 5% (w/v) bovine serum albumin (BSA) dissolved in 1× TBS-T (20 m*M* Tris-base, 0.14*M* NaCl, 0.1% Tween®-20; pH 7.6) for 1 h at room temperature. Membranes were incubated with primary antibodies (anti-CK14 (1:8000 dilution) and anti-CK3 (1:4000 dilution)) diluted in 2% (w/v) BSA dissolved in 1× TBS-T at 4°C overnight. Blots were washed for 45 min in 1× TBS-T before incubation with HRP-conjugated secondary antibodies (anti-mouse (1:8000 dilution) and anti-guinea pig (1:10,000 dilution)) for 2 h at room temperature (RT) with rotation. Proteins were detected on X-ray film using an ECL system; the film was exposed to membranes for 5–10 s to capture protein/ECL signals. CK14 and CK3 expression were detected and blots were stripped (incubation with 2% (w/v) SDS, 5% (v/v) 2-mercaptoethanol in 1× TBS-T at 80°C for 20 min) to remove primary and secondary antibodies, before their total levels were measured using anti-14-3-3ζ antibody (loading control–1:4000 dilution) using the same X-ray film and ECL system. 14-3-3ζ was used as the loading control as it is a protein of low variability comparable to the commonly used loading control GAPDH; we found that GAPDH was difficult to detect clearly in bovine LEC using commercially available antibodies.

#### Collagen IV diffusion from oxidised alginate gels

Collagen IV deposition from oxidised and unmodified alginate gel spheres was measured. Gel spheres were prepared by mixing 1 mg collagen IV into 1 mL alginate solution (final concentration: 1.2% (w/v)), before using a peristaltic pump to dispense alginate solution dropwise into a 102 m*M* CaCl_2_ bath for 10 min. Gels were agitated at 20 rpm and 37°C and the amount of total protein in degraded gel material was quantified using the modified Lowry protein assay.

#### Scanning electron microscopy (SEM) analysis of oxidised alginate gel structure

The internal surfaces of calcium alginate and oxidised calcium alginate gels were examined by SEM. Gels were fixed in 1.25% (v/v) glutaraldehyde and post-fixed for 2 h in 1% (v/v) aqueous osmium tetroxide, after which they were washed in distilled water and passed through a graded ethanol series (50, 70, 90, and 100% (v/v)) before dehydration through critical point drying. Dehydrated gels were fractured to expose internal surfaces, mounted on aluminum stubs and sputter coated with gold before examination using SEM (FEI Quanta FEG 600, UK).

### Rheological measurements

Gel spheres were formed by dispensing 2 mL alginate solutions dropwise into 30 mL CaCl_2_ (102 m*M*). Mechanical testing of gels was achieved by compressing these structures using a TA.XTplus Texture Analyser (Stable Micro Systems, Surrey, UK) with a 5-kg load cell and a P/1KS flat ended stainless steel probe (Stable Micro Systems, Surrey, UK) with a 1 cm^2^ surface area. Measurement of the force was taken, with a trigger force of 0.0005 N, and the yield point of gel spheres (point at which the gels split) was recorded. Force was recorded as the mechanical limit of the gels, described as comparative yield force and 10 measurements of each gel sample were performed.

### Statistical analysis

Unpaired *t* tests were performed using Microsoft Excel. Results are presented as the mean of three individual experiments ± standard error of the mean (S.E.M.), with *P* value < 0.05 considered significant.

## RESULTS AND DISCUSSION

### Formation and structural characterization of oxidised alginate

**FIGURE 1:**
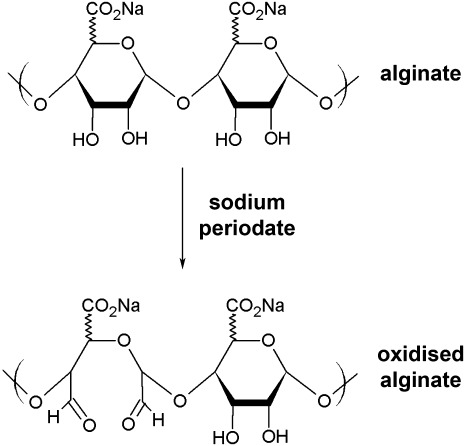
The reaction scheme for oxidation of alginate by sodium periodate.

Alginate was partially oxidized (Fig. 1) to a theoretical extent of 2%, and 5% with sodium periodate following a modified procedure as previously reported.^30^ Oxidation was assessed by measuring levels of sodium periodate after the oxidation reaction. Sodium periodate levels were negligible, indicating that the alginate structure was oxidised.^31^

The average molecular weights (MWs) of native and oxidised alginate were determined using size exclusion chromatography; the MW of 2% oxidised alginate was similar to that of native alginate (99 kDa), while that of 5% oxidised alginate was 68 kDa as would be expected from a more extensive cleavage of the alginate backbone. The extent of oxidation was inversely related to the hydration of the gels (Table I) prepared at the same weight per volume (1.2% w/v) of the native alginate gels; 5% oxidised alginate was significantly (*P* ≤ 0.05) more hydrated than unmodified gels.

**TABLE I:** The Hydration and Stiffness of Unmodified and Oxidized Alginate Gels

Gel type	Mean Hydration (± S.E.M.)	Mean Compressive Modulus (± S.E.M.)
1.2% (w/v) calcium alginate	2.84 ± 0.10	147.02 ± 1.11
2% oxidised 1.2% (w/v) calcium alginate	3.28 ± 0.24	141.21 ± 0.59
5% oxidised 1.2% (w/v) calcium alginate	3.34 ± 0.22	115.17 ± 2.18

Data points represent at least 3 (± S.E.M.) individual measurements of dry and wet weights of gels used to calculate hydration. Data points for compressive moduli (stiffness) represent 10 (± S.E.M.) individual measurements.

The increase in hydration of oxidised alginate-based gels has been previously described. An *in situ* forming hydrogel wound dressing formulated from an oxidised alginate and gelatin matrix, crosslinked together in the presence of borax, was found to have a fluid uptake of 90% of its weight.^24^ This hydrogel was demonstrated to maintain a moist environment over skin wounds whilst absorbing accumulating exudates; the wound bed covered with the hydrogel was filled with epithelium within 2 weeks. Re-epithelialization of the ocular surface is an essential process in corneal wound healing, and hydration is a critical factor for a healthy, functioning cornea. Therefore, oxidised alginate gels may serve as viable wound healing bandages for the damaged cornea that allows for hydration of the corneal surface whilst simultaneously providing an environment for re-epithelialization.

### Corneal epithelial cell viability and phenotype in oxidised alginate gels

In the present study we have begun investigating the possibility that alginate gels are suitable for the development of an artificial LEC niche construct. We assessed the impact of oxidation-induced changes in alginate structure, as well as incorporation of collagen IV, on the viability of corneal epithelial cells and the stem phenotype of limbal epithelial stem cells within these chemically modified matrices.

**FIGURE 2:**
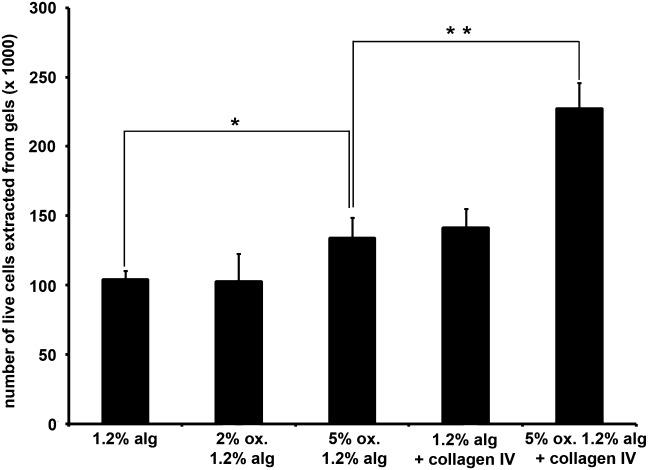
Modification of alginate through oxidation and incorporation of collagen IV enhance the viability of encapsulated corneal epithelial cells. Data points represent the mean number of live cells extracted ± S.E.M (*n* = 3). *(*P* < 0.05) and **(*P* < 0.01) indicate differences between culture conditions.

**FIGURE 3:**
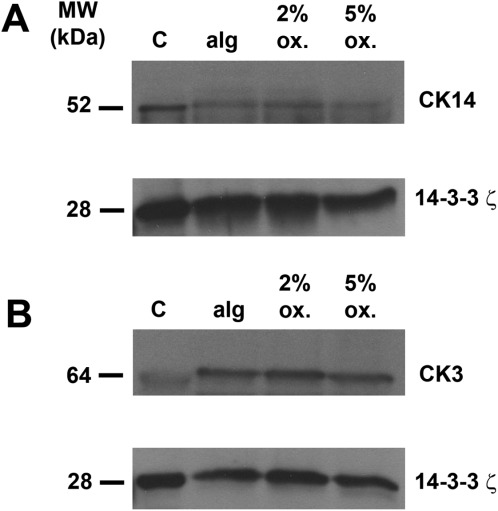
LESC differentiate in oxidised calcium alginate gels. CK14 (A) and CK3 (B) were detected in freshly isolated LEC (control: C) and LEC cultured for 3 days in unmodified and 2 or 5% oxidised alginate gel discs under cell culture conditions by immunoblotting. The 14-3-3ζ was used as a loading control. Blots represent three individual experiments from three different corneoscleral rims.

We observed that modification of alginate gels by oxidation (5%) caused a significant increase (*P* < 0.05) in corneal epithelial cell viability, and the addition of collagen IV into 5% oxidised gels further increased cell viability (*P* < 0.01) (Fig. 2). LEC differentiation (cytokeratin 3–marker of differentiation) in unmodified and oxidised alginate gels was not significantly different (Fig. 3). Although the marker for undifferentiated LEC, cytokeratin 14 (CK14), was expressed in oxidised gels, expression levels were not significantly different between unmodified and oxidised gels. Therefore, oxidised alginate gels may drive the differentiation of progenitor limbal epithelial cells to the same extent as unmodified alginate.

**FIGURE 4:**
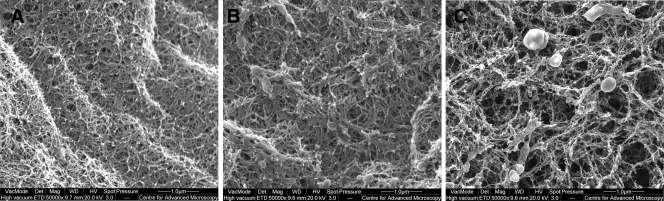
Internal pores in oxidised and unmodified calcium alginate gels. Unmodified (A) and 2% (B) and 5% (C) oxidised calcium alginate HEC gel discs were dehydrated and internal surfaces were examined using SEM. Electron micrographs (×50,000 magnification) are representative of three individual experiments.

The 2% oxidised alginate supported cell viability to a similar extent as unmodified alginate, but presented a similar internal pore size (pore diameter: 0.05–0.1 µm) (Fig. 4) and was significantly less stiff than the unmodified gel (*P* ≤ 0.001) (Table I). By contrast, a significantly greater proportion of the cells encapsulated in 5% oxidised alginate were viable compared to those in unmodified alginate gels, and the 5% oxidised gel displayed internal pores with wider diameters (0.2–0.8 µm), and significantly lower stiffness than unmodified alginate. It is likely that the metabolic activity and viability of corneal epithelial cells released from oxidised alginate gels correlate, as we have previously reported similar results with unmodified alginate gels.^8^ We have also shown that live epithelial cells (viability assessed by Trypan blue exclusion) released from alginate gels are able to grow and form into colonies.^8^ This indicates that live cells released from oxidised alginate gels remain functional. In our reported studies some alginate gels allowed 100% recovery of encapsulated live cells.^8^ Therefore it is possible that as full recovery of encapsulated live cells was not achieved in the present study, a population of those cells underwent apoptosis. These data indicate that increased pore size conferred by oxidation is a greater determinant of the increase in cell viability, than decreased stiffness.

We also investigated the diffusion of collagen IV from oxidised alginate gels, to begin examining the potential stability of these constructs under physiological conditions. The concentration of collagen IV lost from 5% oxidised alginate gels was significantly greater after 48 h than 24 h, but there was no difference in protein loss compared to unmodified gels at either time point (Table II). Our data suggest that ECM molecules may become entrapped within alginate gels or, in the case of oxidized alginate, there is the potential of covalent incorporation of the protein by reaction of a free amine with aldehydes in the alginate. It is unlikely that collagen IV has become covalently bound to oxidised alginate in the present study as the concentration of protein released is similar between oxidised and unmodified alginate.

**TABLE II:** Diffusion of Collagen IV from Oxidised Alginate Gels

Gel Type	Concentration of Diffused Collagen IV (µg mL^−1^) (± S.E.M.)
1.2% (w/v) calcium alginate + collagen IV – 24 h	177.42 ± 0.010
5% oxidised 1.2% (w/v) calcium alginate + collagen IV – 24 h	177.42 ± 0.008
1.2% (w/v) calcium alginate + collagen IV – 48 h	594.87 ± 0.006
5% oxidised 1.2% (w/v) calcium alginate + collagen IV – 48 h	599.04 ± 0.009

Data represent the mean ± S.E.M. amount (µg mL^−1^) of collagen IV protein released from unmodified and 5% oxidised calcium alginate gels (*n* = 3).

Previous studies describe cell viability profiles in oxidised alginate gels that are in line with our findings. Hepatocytes encapsulated in periodate-oxidised alginate (containing crosslinked gelatin in the presence of sodium tetraborate), maintained their characteristic morphology after 4 weeks in this matrix.^19^ These cells also remained functional, retaining their albumin producing ability for up to 2 weeks following encapsulation. Oxidised and RGD-grafted alginate microspheres loaded with human osteoprogenitors from bone marrow mesenchymal stem cells with or without human umbilical vein endothelial cells increased mineralization within bone defects in animal models.^23^ NH3T3 fibroblasts immobilized in a composite *N*,*O*-carboxymethyl chitosan/oxidized alginate hydrogel were shown to spread and grow inside this gel.^20^ A similar composite gel based on hydroxypropyl chitosan and periodate-oxidised sodium alginate dialdehyde was reported as a potential scaffold for reconstruction of the corneal endothelium.^18^ Corneal endothelial cells encapsulated in the composite gel system on Descemet's membranes survived and retained their normal morphology. Therefore, corneal tissue engineering approaches may be taken using oxidised alginate gels as a basis.

Certainly, these matrices have been used together with human adipose stem cells (hADSC) and MSC for engineering adipose^22^ and osteogenic tissue,^21^ respectively. hADSC were differentiated into adipogenic cells and encapsulated into alginate gels modified by partial periodate oxidation.^22^ hADSC laden gels were subcutaneously injected into the chest wall of male nude mice. Newly generated adipose tissue was produced after 10 weeks in all experimental mice. Oxidised alginate microspheres were also shown to direct the differentiation of MSC to osteogenic and adipogenic tissues.^21^ Encapsulated MSC remained viable *in vitro* and both osteo-differentiated and adipo-differentiated after 4 weeks of culturing in induction media. Similarly, findings of the present study show that LESC differentiate in oxidised alginate gels.

Studies have also described oxidised alginate gels containing proteins or peptide motifs for wound healing applications.^32^,^33^ In the present study, we incorporated collagen IV into alginate gels to investigate the effect of ECM proteins in addition to oxidation on the viability of encapsulated cells. Periodate-oxidised alginate rapidly crosslinks proteins (e.g., gelatin) in the presence of borax (tetrasodium borate) to produce *in situ* forming hydrogels that are biocompatible and biodegradable. These hydrogels were demonstrated as efficient wound healing dressings on full thickness wounds in a rat model; within 2 weeks of application the wound covered with gel was completely filled with new epithelium.^24^ Periodate-oxidised alginate/gelatin wound dressings have also been applied for the delivery of molecules that promote wound healing. Dibutyryl cyclic adenosine monophosphate (DBcAMP), a lipophilic analog of cAMP was incorporated into this gel construct.^33^ Evaluation of the efficacy of the dressing on full thickness wounds demonstrated complete re-epithelialization of the wound within 10 days. Re-epithelialization of the cornea may be possible using *in situ* formed oxidised alginate gels containing gelatin.

In the present study, we hypothesize that the differences in viability observed between native and oxidised alginate gels may be due to increased porosity caused by oxidation-induced changes in gel mass, as we have previously reported that increasing the porosity of alginate gels increases the viability of encapsulated CEC.^8^ We observed that the porosity of oxidised gels is greater than that of native gels, and that gel stiffness decreases with increases in the extent of oxidation. We associated the degree of oxidation, porosity and stiffness to a discrete level of cell viability. It may be possible to investigate the effect of alginate gel stiffness on the phenotype and viability of encapsulated LEC. Alginate has a backbone of rigid α-l-guluronate and more flexible β-d-mannuronate; the chain stiffness and steric hindrance of polyguluronate molecules from alginate can be regulated by partial oxidation.^34^ The significantly reduced stiffness we measured is likely due to chemical oxidation and a lowering of molecular weight.

The stiffness of oxidised alginate gels is an important property of these gels that is currently being exploited in the biomedical field. Biodegradable, partially crosslinked alginate scaffolds with shape-memory properties were previously fabricated for minimally invasive surgical applications. These were constructed by mixing together high and low molecular weight partially oxidized alginate modified with RGD peptides and performing covalent crosslinking using carbodiimide chemistry. The resulting scaffold was compressible 11-fold and returned to its original shape when rehydrated.^35^ Other studies demonstrated *in situ* gelable hydrogels based on oxidised alginate and gelatin^36^ or teleostean^37^ with elastic moduli that can be controlled by adjusting the oxidation degree of alginate and the weight ratio of gelatin and teleostean.

Another important physical property conferred by oxidation is the controlled degradation of alginate gels; alginate gels tend to disintegrate in electrolyte solutions. Alginate oxidised to a low degree (∼5%), was reported to degrade at a rate dependent on pH and temperature—the gel degraded within 9 days in PBS solution.^30^ Interestingly, studies have shown that the degradation of oxidised alginate can be regulated. Poly(acrylamide-*co*-hydrazide) (PAH), a multi-functional crosslinking molecule, or adipic acid dihydrazide (AAD), a bi-functional crosslinking molecule, were used to crosslink oxidised alginate gels.^38^ PAG/PAH hydrogels showed higher mechanical stiffness before degradation and degraded more slowly than PAG/AAD gels, at the same concentration of crosslinking functional groups. The enhanced mechanical stiffness and prolonged degradation behavior could be attributed to the multiple attachment points of PAH in the gel at the same concentration of functional groups. Another method for controlling alginate gel degradation employed methacrylation that changes uronate residue conformations to an open chain adduct, making them more vulnerable to hydrolysis.^39^ This modification allows the degradation profiles of alginate matrices to be tunable by the degree of oxidation.

Taken together, we conclude that the findings of the present study describe novel avenues for the culture of LEC that can form the foundation of alginate-based niche constructs focusing on chemical oxidation and incorporation of corneal ECM proteins.

## CONCLUSIONS

Our study concludes that corneal epithelial cells can be maintained in porous, low modulus, oxidised alginate gels containing collagen IV, with predictable diffusion rates for ECM proteins in physiological environments. Oxidation of alginate gels may be employed as a means to control the mass transfer of biomolecules from immobilized cells whilst allowing controlled degradation of these scaffolds to release therapeutic cells. This may be an important future development in the use of alginate constructs for corneal cell delivery and culture. Therapy for LSCD may be refined (i.e., variability in treatment outcomes could be eliminated), through the use of these multi-functional hydrogel niche-like devices. The degree of oxidation may even be tuned to control corneal cell function and LESC fate. We have acquired initial components of a toolbox that will allow control of individual aspects of LESC function, by altering physical properties of gels used to immobilize these cells before their therapeutic delivery.
